# KT-YOLO: A multi-convolution kernel collaboration model for dense Hu sheep behavior detection

**DOI:** 10.1371/journal.pone.0349267

**Published:** 2026-05-18

**Authors:** Suoxiang Zhang, Hongrui Chang, Zhonghong Wu, Guoxin Wu, Ronghua Ji

**Affiliations:** 1 College of Information and Electrical Engineering, China Agricultural University, Beijing, China; 2 College of Animal Science and Technology, China Agricultural University, Beijing, China; Macau University of Science and Technology, MACAO

## Abstract

Computer vision has been extensively applied to sheep behavior detection in recent years. However, the dense distribution of Hu sheep poses detection challenges, while imbalanced behavioral categories in datasets affect classification accuracy for detection tasks in intensive farming scenarios, resulting in high misclassification rates. Current models often rely on over-parameterization to achieve satisfactory detection performance, which increases computational burden and limits practical deployment. To address these challenges, this study introduces the Hu Sheep Behavior Dataset (HSBD), specifically designed for intensive farming environments. The dataset comprises 280 images capturing four behaviors across 6,766 Hu sheep: standing, lying, eating, and drinking. Building upon this foundation, we developed the KT-YOLO model, which utilizes a novel Kernel-Team Fusion (KTF) method to enhance the YOLOv8n detection framework. By employing four different convolution kernel sizes, this method effectively captures multi-scale features and addresses Hu sheep occlusion challenges. To mitigate accuracy degradation caused by dataset imbalance, KT-YOLO incorporates a SlideLoss function during classification, effectively addressing this challenge. Comparative experiments demonstrate that KT-YOLO achieved a mean Average Precision (mAP50) of 86.4%, representing a 6.3 percentage point improvement over YOLOv8n, with SlideLoss contributing an additional 1 percentage point improvement. Further comparison with YOLOv13n demonstrates KT-YOLO’s superior performance in dense Hu sheep behavior detection. By introducing HSBD and developing the innovative KT-YOLO, this study significantly enhances both accuracy and efficiency of dense Hu sheep behavior detection, demonstrating the potential and practical value of deep learning technologies in intensive farming environments.

## Introduction

The growing global population and increasing demand for animal protein products pose significant challenges to modern livestock farming [1]. To address these challenges, improving breeding efficiency, reducing operational costs, and enhancing animal welfare are key objectives for contemporary livestock farming.

Automated animal behavior detection has become critical for achieving these objectives, as behavioral patterns provide early indicators of health problems, feeding efficiency, and environmental stress. This enables continuous assessment of animal welfare status and timely interventions for disease prevention and production optimization. Traditional manual observation methods [2] are labor-intensive, time-consuming, and increase zoonotic disease transmission risks through direct human-animal contact. Therefore, developing automated detection technologies has become increasingly critical for modern livestock management.

Automated animal behavior detection approaches can be categorized into invasive and non-invasive methods [3]. Invasive methods include embedded sensor technologies, while non-invasive methods encompass computer vision technologies. Automated animal behavior detection based on embedded sensor technologies [4] can improve breeding efficiency and provide critical insights for animal health assessment and behavioral analysis.

While embedded sensors demonstrate clear advantages in data accuracy, their invasive nature may interfere with animals’ natural behaviors, and associated costs remain prohibitively high for many farms [5]. These limitations have prompted researchers to seek more economically viable alternative technologies.

In recent years, detection methods that combine image with deep learning technologies have been extensively explored [6,7]. This approach offers numerous advantages, being non-invasive, cost-effective, and highly efficient. It is particularly suitable for detecting daily behaviors such as lying [8], eating [9], drinking [10], and standing [11], as well as specific behaviors like parturition [12], lactation [13], aggression [14], and mounting [15].

Significant advances in computer vision technology and growing demand for real-time detection capabilities have driven the development of efficient architectures such as YOLO [16], SSD [17], and MobileNet [18]. These architectures have substantially improved animal behavior detection efficiency. However, intensive farming presents distinct challenges, with high-density scenarios characterized by dataset scarcity, severe target occlusion, and high computational costs.

Research progress in animal behavior detection under realistic high-density intensive farming conditions remains limited, primarily due to the lack of appropriate datasets. Previous sheep behavior datasets [19,20] typically contain at most 13 sheep per image, whereas dense scenarios in intensive farming can contain 30–40 sheep within a single camera field of view based on actual commercial breeding densities. This high-density scenario represents more than a quantitative increase—it introduces a significant shift in detection tasks from relatively independent individual detection to scene understanding tasks requiring complex group interaction processing.

Existing behavior detection models, predominantly designed for sparse scenarios, face significant difficulties when processing dense scenarios. Extensive mutual occlusion makes it hard to obtain complete individual contours, directly affecting accurate bounding box localization [21]. Additionally, dense farming scenarios present complex feature extraction challenges. The aggregation of animals with similar postures leads to feature confusion under realistic intensive farming conditions [22], particularly when distinguishing subtle behavioral differences between adjacent individuals, such as differentiating between low-head standing and eating behaviors. Moreover, single-scale feature extraction cannot simultaneously capture individual local details and group-level global layout information [23]. Furthermore, livestock animals’ behavioral patterns naturally result in class imbalance problems. Due to animals’ natural time allocation, certain behaviors such as drinking occur significantly less frequently than others like standing or lying, creating an inherent imbalance that affects model robustness and detection accuracy. Finally, excessive computational requirements hinder deployment on resource-constrained edge devices in agricultural environments [24]. These factors collectively limit the application of previous detection architectures in dense farming scenarios.

To address these challenges, this study makes the following contributions

First, design and construction of the Hu Sheep Behavior Dataset (HSBD) specifically for dense farming scenarios, comprising 6,766 Hu sheep instances with up to 36 Hu sheep per image. Second, development of the Kernel-Team Fusion (KTF) method, employing four different convolution kernel sizes (1×1, 3×3, 5×5, 7×7) to capture multi-scale features, effectively addressing occlusion challenges in dense scenarios while maintaining computational efficiency. Third, integration of the SlideLoss [25–30] loss function to address the inherent class imbalance stemming from natural animal behavioral patterns, enhancing model robustness and detection accuracy.

To validate our approach, we conducted comprehensive performance comparisons with various representative models, demonstrating superior accuracy-efficiency trade-offs for dense Hu sheep behavior detection tasks. These contributions collectively advance the field of automated animal behavior detection, providing a technical foundation for future deployment in intensive farming environments.

## Materials and methods

### Dataset construction

HSBD was collected at Anxin Animal Husbandry Co., Ltd. in Bozhou City, Anhui Province, China, during two periods: August 22–27, 2023 (temperature: 29.52 ± 2.11°C, humidity: 80.65 ± 7.07%RH) and January 8–17, 2024 (temperature: 4.39 ± 2.47°C, humidity: 68.15 ± 8.38%RH). Data collection was conducted in a Hu sheep barn consisting of 60 pens, each measuring 80 meters in length, 18 meters in width, and 4 meters in height. The stocking density was approximately 0.63 sheep per square meter. Feed and water were provided through feeding troughs and automatic water dispensers, allowing Hu sheep free access to food and water throughout the experimental period.

During data collection, RGB cameras (DH-IPC-HFW2433F-ZAS, Dahua Technology Co., Ltd., Hangzhou, China) were deployed to record videos across multiple Hu sheep pens. Video clips were stored in MP4 format on 512GB memory cards with a resolution of 1920×1080 pixels and a frame rate of 25 frames per second. Since Hu sheep frequently maintain the same position for extended periods during farming processes, frames were extracted at 180-frame intervals from recorded videos for subsequent behavioral analysis. The definitions of Hu sheep behaviors are presented in Table 1, which were established following comprehensive analysis and discussion with livestock experts.

**Table 1:** The definitions of Hu sheep behavior categories in HSBD.

Behavioral category	Definitions
Standing	Maintaining an upright posture without other specific actions
Lying	Sitting on the floor with the body trunk in close proximity to the ground
Eating	Standing with the head in the feeding trough
Drinking	Standing with the head in the automatic drinking trough

**Fig 1:**
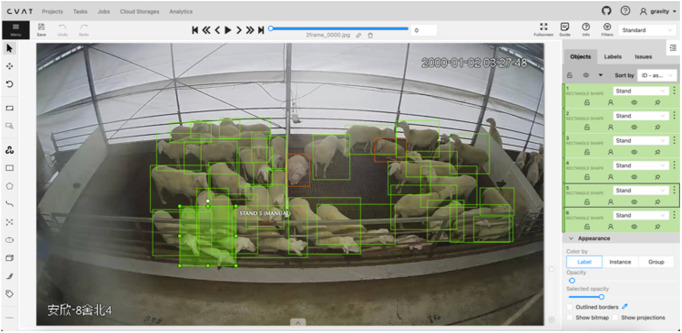
Annotation example of Hu sheep behaviors in HSBD using CVAT platform. Representative image showing the annotation process with bounding boxes marking different sheep behaviors: red boxes indicate standing, pink boxes indicate lying, orange boxes indicate eating, and yellow boxes indicate drinking behaviors.

HSBD was annotated using the CVAT platform, with each instance labeled using bounding boxes and corresponding behavioral categories (Fig 1).

**Fig 2:**
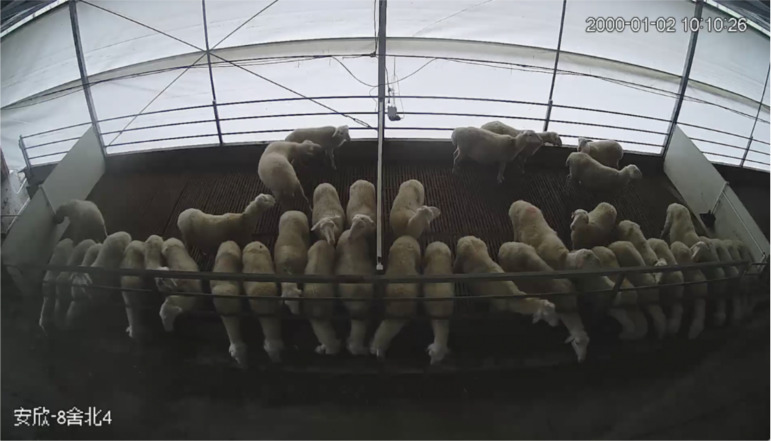
Representative dense distribution scenario in HSBD. Dense distribution scenario from HSBD showing up to 36 Hu sheep per image in intensive farming conditions, characterized by substantial mutual occlusion among individuals.

We conducted quantitative comparisons with the existing sheep behavior dataset [19]. While the existing dataset contains 3,874 instances with at most 13 sheep per image, HSBD comprises 280 images with 6,766 total instances and up to 36 sheep per image (Fig 2), specifically targeting high-density scenarios characteristic of intensive farming.

For experimental validation, the dataset was partitioned at the image level into training and testing sets comprising 210 and 70 images, respectively (approximately 75%/25% by image count). This image-level partitioning ensures that all instances from a given image appear exclusively in either the training or testing set, thereby preventing data leakage that could arise from instance-level splitting where correlated instances from the same image might appear in both sets. The resulting instance distribution corresponds to approximately 80% and 20% of the total annotated instances for training and testing, respectively. The training set comprised mixed daytime and nighttime instances to develop a unified model capable of operating under varying lighting conditions. The test set was strategically divided by capture time to evaluate model performance across different illumination scenarios. The distribution of different behaviors between training and testing sets is presented in Table 2.

**Table 2:** The distribution of different behaviors in dataset.

Behavioral categories	Train	Test
Standing	2725	813
Lying	859	248
Eating	1706	257
Drinking	125	33
Total	5415	1351

Notably, drinking behavior was predominantly observed during daytime periods, consistent with the established circadian rhythm of water intake in sheep [31,32]. During systematic review of all nocturnal video recordings, only 1–2 drinking instances were identified, confirming the rarity of nocturnal drinking behavior. However, these instances were excluded from annotation due to severely degraded image quality under low-illumination conditions, as the drinking posture—with the head inserted into peripherally located water dispensers—requires finer spatial discrimination than standing or lying postures under equivalent low-light conditions. Consequently, the 158 drinking instances in HSBD (125 training, 33 testing) are entirely from daytime observations.

### KT-YOLO architecture

Developing effective detection models for dense Hu sheep farming requires careful consideration of the fundamental trade-off between detection accuracy and computational efficiency. We selected YOLOv8n [33] as our baseline architecture, whose modular design provides an ideal foundation for integrating our proposed multi-scale feature fusion methodology. This integration maintains the real-time processing capabilities essential for practical deployment.

**Fig 3:**
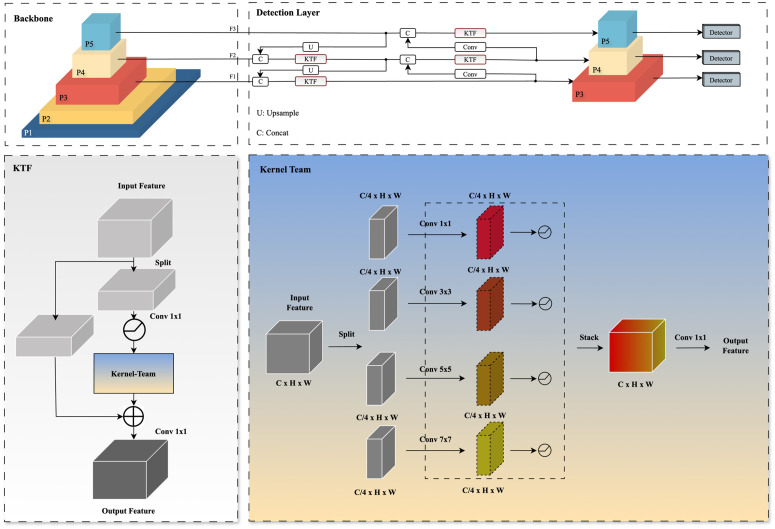
KT-YOLO architecture. The overall architecture showing the backbone network with KTF modules and the detection layer. The model processes input images through multiple KTF modules that employ different convolution kernel sizes for multi-scale feature extraction.

Based on the YOLOv8n foundation architecture, this study presents KT-YOLO, a model specifically engineered for detecting Hu sheep behavior in dense farming scenarios. KT-YOLO employs convolutional layers with different kernel sizes to process image features at multiple granularities, achieving superior detection performance. KT-YOLO comprises a backbone network and detection layers, with its specific architecture illustrated in Fig 3. We designed a feature fusion module called the Kernel-Team Fusion (KTF) module, which efficiently performs multi-scale feature extraction and fusion throughout the backbone network. Since dense farming scenarios typically present class imbalance in Hu sheep behavior data collection, we adopted the SlideLoss loss function in the detection layer to effectively mitigate the degradation of robustness and accuracy caused by class imbalance.

### KT-YOLO backbone

**Fig 4:**
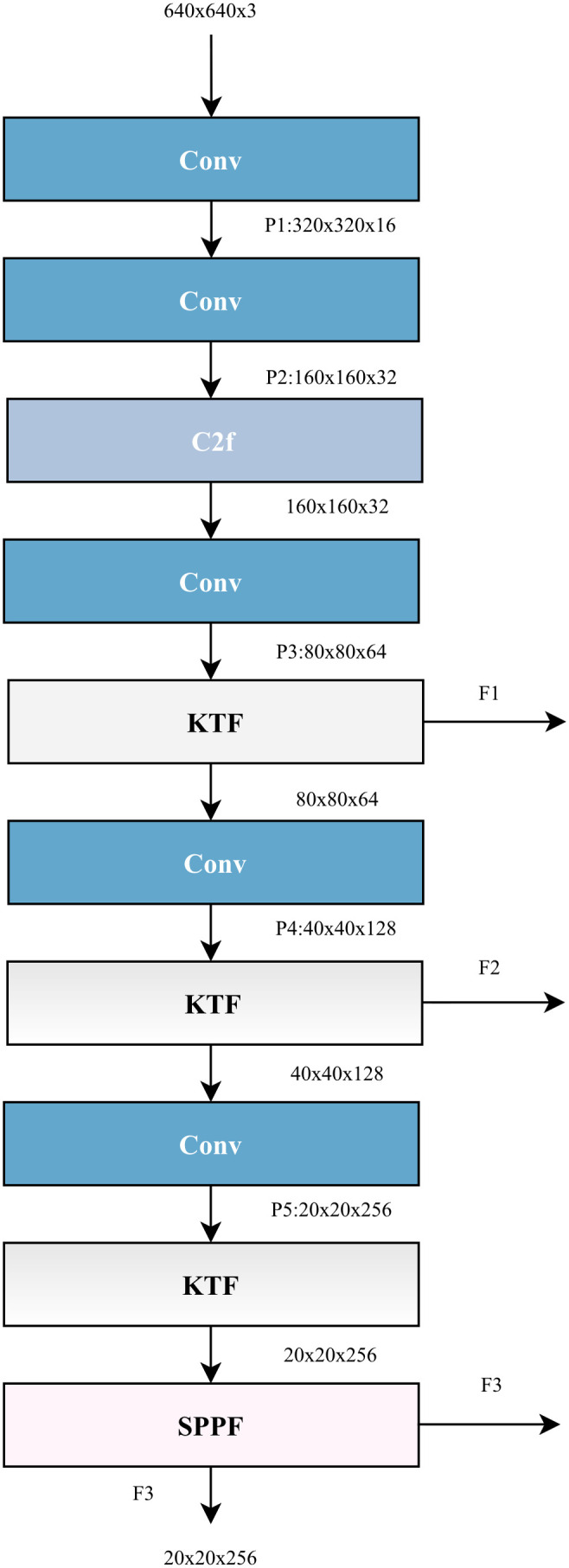
KT-YOLO Backbone architecture. Detailed view of the backbone network showing the arrangement of KTF modules, C2F layers, and SPPF components for efficient feature extraction.

The KT-YOLO backbone network, illustrated in Fig 4, incorporates convolutional layers, feature fusion computational layers (KTF and C2F), and Spatial Pyramid Pooling Feature (SPPF) layers. C2F represents Cross Stage Partial Bottleneck with 2 convolutions.

The Kernel-Team Fusion (KTF) network architecture effectively enhances dense Hu sheep behavior feature extraction by combining traditional convolutional structures with innovative multi-scale convolution fusion techniques. As depicted in Fig 3, input features undergo channel splitting, where a portion of the features is processed through convolutional layers to reduce dimensionality, decreasing computational complexity while preserving essential information. The remaining portion is concatenated with the results of kernel-team computation. This design effectively mitigates the gradient vanishing problem inherent in deep networks, enabling practical training of deeper network architectures while accelerating model training speed and enhancing overall model performance. Multi-scale feature processing is conducted through the kernel-team approach, enabling the model to more effectively capture and fuse fine-grained information across different spatial scales.

The core of the kernel-team is a multi-scale convolution processing module that employs different sizes of convolution kernels (1×1, 3×3, 5×5, 7×7) to process input features in parallel. This approach enables the network to capture information at different scales while reducing computational load through channel splitting, with different kernel sizes providing complementary feature representations. Each kernel size receives an evenly distributed number of channels from the original input features, with a minimum channel constraint of 8 ensuring that each partition retains sufficient feature representation capability. Finally, these multi-scale features are integrated through 1×1 convolution, effectively combining and reorganizing inter-channel features to enhance the model’s feature representation capability. The specific convolution operations can be represented by Eq (1), Eq (2), and Eq (3).


$$\begin{array}{c} f_{i}(x)=\sigma (Conv_{Kernel_{j}}(InputFeature_{i})) \end{array}$$ (1)

$$\begin{array}{c} S=Stack(f_{1}(x)\cdots f_{i}(x)) \end{array}$$ (2)

$$\begin{array}{c} OutputFeature=Conv_{1\times 1}(S) \end{array}$$ (3)

where σ represents the SiLU activation function, *i* denotes the feature partition index, *j* corresponds to the kernel size (1×1, 3×3, 5×5, or 7×7), and Stack(·) concatenates features along the channel dimension.

### KT-YOLO detection layer

**Fig 5:**
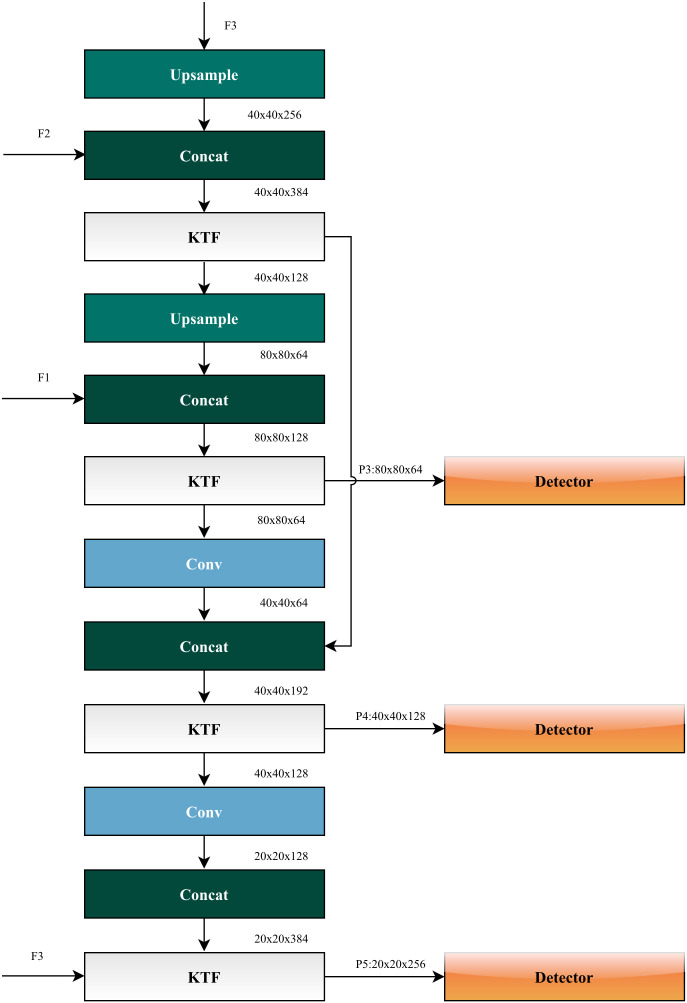
KT-YOLO Detection Layer architecture. The detection layer processes multi-scale features from the backbone network and generates three detection heads for different scale object detection.

The KT-YOLO detection layer is specifically designed for efficient feature extraction, classification, and detection, with its detailed architecture illustrated in Fig 5.

The KT-YOLO backbone network extracts multi-scale features F1 (80 × 80 × 64), F2 (40 × 40 × 128), and F3 (20 × 20 × 256), which are forwarded to the KT-YOLO detection layer. This architecture utilizes 3 × 3 convolutional layers, upsampling layers, and KTF feature fusion layers to generate three multi-scale detection heads. Following the extraction and fusion of multi-scale behavioral features, the KT-YOLO network performs object classification and bounding box regression through a decoupled head design. Assuming a standard 640 × 640 input resolution, the spatial dimensions of the prediction grids produced by the three detection heads are 80 × 80, 40 × 40, and 20 × 20, corresponding to downsampling strides of 8, 16, and 32, respectively. This multi-scale prediction strategy ensures accurate detection across varying Hu sheep sizes and occlusion levels.

Dense Hu sheep farming scenarios present inherent challenges in object detection, particularly regarding the imbalance of sample difficulty. To address this, SlideLoss is adopted as the classification loss function. SlideLoss specifically targets difficult samples by dynamically adjusting loss weights based on prediction quality (IoU), as expressed in Eq (4).


$$\begin{array}{c} f(x)=\left\{ \begin{array}{cc} 1 & x\leq \mu -0.1\\ e^{1-\mu } & \mu -0.1<x<\mu \\ e^{1-x} & x\geq \mu \end{array}\right. \end{array}$$ (4)

where *x* represents the IoU value, μ is the difficulty threshold parameter, and 0.1 determines the transition interval width.

In the SlideLoss formulation, the weighting function *f*(*x*) (where *x* denotes the IoU value as defined in Eq (4)) is applied to the standard Binary Cross-Entropy (BCE) loss as a sample-wise multiplier. The final classification loss is computed as:


$$\begin{array}{c} {ℒ}_{cls}=f(x)\cdot {ℒ}_{BCE} \end{array}$$ (5)

where ${ℒ}_{BCE}$ is the standard BCE loss. The threshold μ is set to the mean IoU of the training set. Mathematically, samples with low IoU (x≤μ−0.1) retain a base weight of 1. To prioritize difficult boundary samples, those in the transition interval [μ−0.1,μ) receive an elevated constant weight of $e^{1-\mu }$. For well-localized samples (x≥μ), the weight decays exponentially ($e^{1-x}$), down-weighting easy examples. This mechanism effectively shifts the training focus to hard-to-classify samples, thereby enhancing the model’s generalization capability in dense sheep detection tasks.

## Results

This section describes our experimental setup and evaluation metrics. We evaluate KT-YOLO by comparing it with representative detection techniques and systematically analyze its performance on different Hu sheep behaviors from both quantitative and qualitative perspectives.

### Experimental settings

The experimental environment for this study used an Ubuntu 16.04 operating system, equipped with an Intel(R) Xeon(R) CPU E5-2686 v4 processor, a 3080 Ti-12G GPU, and 12GB of RAM. The hyperparameters for model training were as follows: the training optimizer was SGD, with a momentum of 0.937, the batch size was 16, a weight decay set to 0.0005, and a learning rate of 0.01. The entire network was trained for 500 epochs. EarlyStopping was employed with a patience setting of 50. For a fair comparison, each model was trained with the same parameters.

### Evaluation metrics

The evaluation of the model involves using Precision, Recall, Average Precision (AP), and Mean Average Precision (mAP) metrics. The specific calculation formulas for each metric are as follows:


$$\begin{array}{c} Precision=\frac{TP}{TP+FP}\times 100\% \end{array}$$ (6)

$$\begin{array}{c} Recall=\frac{TP}{TP+FN}\times 100\% \end{array}$$ (7)

$$\begin{array}{c} AP=\int_{0}^{1}Precision(Recall)dR \end{array}$$ (8)

$$\begin{array}{c} mAP=\frac{\sum_{i=1}^{n}AP_{i}}{n} \end{array}$$ (9)

The model was evaluated using Precision, Recall, Average Precision (AP), and Mean Average Precision (mAP). True Positives (TP) refer to correctly identified objects, False Positives (FP) to incorrectly identified objects, and False Negatives (FN) to missed objects. AP measures the area under the Precision-Recall curve, while mAP averages AP across all classes. Additionally, model parameters (Params) and Giga Floating Point Operations Per Second (GFLOPS) were used to assess the model’s suitability for mobile applications, with higher values indicating greater computational demands.

### Comparative experiments

We conducted comprehensive evaluations to assess the effectiveness of KT-YOLO. We compared it with multiple representative detection methods that adopt different technical approaches. This enables validation of our method’s advantages from multiple perspectives.

We evaluated four mainstream detection methods to assess their performance on specialized dense detection tasks (Table 3). These methods represent different technological development directions and demonstrate representativeness in their respective technical approaches but exhibit significant performance differences on dense Hu sheep detection tasks. The experimental parameters used were identical to those employed for KT-YOLO.

**Table 3:** Performance comparison of different detection methods.

Method	mAP50(%)	mAP50:95(%)	FPS	Params(MB)	GFLOPs
Fast R-CNN	49.6	30.2	21.76	141.89	34.47
SwinTransformer	72.6	39.7	19.74	123.88	38.23
EfficientDet	80.4	54.4	27.55	12.09	8.8
YOLOv13n	73.5	41.4	47.2	5.4	6.4
**KT-YOLO**	**86.4**	**60.8**	**85.3**	**5.8**	**8.5**

Fast R-CNN [34] represents classical two-stage detection frameworks, theoretically providing more precise detection through candidate region generation and refined classification strategies. However, it achieved only 49.6% mAP50 on our dataset with a substantial model overhead of 141.89MB and high computational demand of 34.47 GFLOPs, indicating fundamental challenges faced by traditional detection frameworks when handling high-density targets. Swin Transformer [35] represents modern detection methods based on attention mechanisms, capturing global dependencies through self-attention mechanisms to facilitate understanding of complex spatial relationships between individuals in dense scenes. However, it achieved only 72.6% mAP50 on the dataset with a model size of 123.88MB and computational load of 38.23 GFLOPs, creating efficiency challenges in practical applications. EfficientDet [36] represents multi-scale detection optimization work, achieving a balance between detection accuracy and efficiency through compound scaling and bidirectional feature pyramid networks (BiFPN), performing optimally among the four methods with 80.4% accuracy. YOLOv13n [37] represents the latest advancement in lightweight detection architectures, achieving 73.5% mAP50 with excellent computational efficiency (5.4MB, 6.4 GFLOPs), but still falling short of the requirements for dense detection scenarios.

When these general methods are compared with KT-YOLO, the performance gap becomes apparent. EfficientDet also adopts multi-scale feature fusion strategies; however, its BiFPN design is primarily optimized for general detection tasks and demonstrates limitations when confronting the specific challenges of dense Hu sheep scenarios. There is a 6 percentage point gap compared to KT-YOLO’s 86.4% mAP50. More critically, EfficientDet’s model size (12.09MB) is approximately twice that of KT-YOLO (5.8MB) while also demonstrating lower computational efficiency. Similarly, YOLOv13n, despite its computational advantages, shows a 12.9 percentage point accuracy gap compared to KT-YOLO. These results indicate that while general detection methods have advantages in their respective domains, the absence of targeted design significantly impacts their performance when addressing high-density, heavily occluded specialized detection scenarios, particularly evident in balancing computational efficiency and detection accuracy.

To further evaluate localization quality beyond the IoU = 0.5 threshold, we report mAP50:95 across all methods. KT-YOLO achieved the highest mAP50:95 of 60.8%, outperforming EfficientDet (54.4%) and YOLOv13n (41.4%), confirming that the multi-scale kernel-team fusion mechanism improves not only coarse detection but also precise bounding box localization under heavy occlusion conditions. Regarding inference efficiency, KT-YOLO achieved 85.3 FPS, substantially exceeding the real-time threshold (30 FPS) and outperforming all other evaluated methods in Table 3, demonstrating practical viability for continuous monitoring deployment.

## Ablation studies

### Overall architecture analysis

We conducted direct comparisons between KT-YOLO and its baseline architecture YOLOv8n (Table 4) to quantify the improvement effects of our proposed overall architecture.

**Table 4:** Comparative experimental results of YOLOv8n and KT-YOLO.

Method	mAP50(%)	mAP50:95(%)	FPS	Params(MB)	GFLOPs
YOLOv8n	80.1	55.1	**93.8**	6.1	8.7
**KT-YOLO**	**86.4**	**60.8**	85.3	**5.8**	**8.5**

This comparison represents the performance differential following complete removal of all improved components (KTF module and SlideLoss), validating the overall effectiveness of the proposed architecture. Experimental results demonstrate that KT-YOLO achieved a significant 6.3 percentage point improvement in mAP50 compared to YOLOv8n (86.4% vs. 80.1%) while simultaneously enhancing model efficiency—model size decreased from 6.1MB to 5.8MB and computational requirements reduced from 8.7 GFLOPs to 8.5 GFLOPs. This result substantiates that our architectural design improves detection accuracy and optimizes computational efficiency, providing favorable conditions for deployment in resource-constrained agricultural environments.

The mAP50:95 improvement from 55.1% to 60.8% (+5.7 percentage points) demonstrates that KT-YOLO enhances localization precision at stricter IoU thresholds, indicating more accurate bounding box predictions particularly important in dense scenarios where tight localization is essential for distinguishing adjacent individuals. The architectural modifications introduce a modest reduction in inference speed (85.3 FPS vs. 93.8 FPS for YOLOv8n), attributable to the parallel multi-kernel computation in the KTF module. However, this 9% speed reduction is accompanied by a 6.3 percentage point gain in mAP50 and a 5.7 percentage point gain in mAP50:95, representing a favorable accuracy-efficiency trade-off for practical deployment where both speeds well exceed real-time requirements.

### KTF component analysis

We conducted systematic ablation experiments (Table 5) to comprehensively understand the specific contributions of each component within the Kernel-Team Fusion (KTF) mechanism. The experimental results revealed progressive improvements from multi-scale feature fusion. Individual convolution kernels possess specific advantages at their respective scales; however, no single scale can adequately address the complexity of dense Hu sheep detection scenarios.

**Table 5:** Ablation Study of KTF Components.

Kernel configuration	mAP50(%)
1×1	81.5
3×3	82.3
5×5	81.8
7×7	81.2
3×3 + 5×5	84.4
1×1 + 3×3 + 5×5	84.7
**All**	**86.4**

Among them, the 3×3 kernel demonstrated optimal performance when used individually (82.3% mAP50), correlating with its balanced capability in capturing medium-scale features. Multi-kernel combination experiments demonstrated obvious cumulative effects: dual-kernel configuration (3×3 + 5×5) achieved 84.4% mAP50, triple-kernel configuration (1×1 + 3×3 + 5×5) further improved to 84.7% mAP50, and the complete four-kernel configuration ultimately achieved 86.4% mAP50. This progressive improvement pattern substantiates the importance of complementary features provided by different scale convolution kernels for dense detection tasks. Smaller kernels (1×1, 3×3) excel at capturing fine posture features necessary for distinguishing adjacent individuals, while larger kernels (5×5, 7×7) provide wide-area spatial context information essential for parsing complex occlusion relationships.

### Loss function analysis

Our dataset analysis revealed significant imbalance phenomena between different behavioral categories in dense farming environments, in addition to architectural innovation. This may lead to model training bias toward high-frequency behavioral categories. To evaluate the specific effects of SlideLoss in alleviating class imbalance problems, we designed comparative experiments (Table 6) to isolate the independent contribution of the loss function.

**Table 6:** Comparative experimental results of different loss functions.

Method	Loss Function	mAP50(%)
YOLOv8n	BCE	80.1
YOLOv8n	SlideLoss	84.7
KT-YOLO	BCE	85.4
**KT-YOLO**	**SlideLoss**	**86.4**

Experimental results demonstrate that the introduction of SlideLoss yielded significant performance improvements for both architectures. YOLOv8n’s mAP50 substantially improved from 80.1% to 84.7% (+4.6 percentage points), while within the KT-YOLO architecture, SlideLoss further enhanced performance from 85.4% to 86.4% (+1.0 percentage point). SlideLoss demonstrated more pronounced improvement in YOLOv8n, indicating that when a model’s feature extraction capability is limited, balanced loss function design can exert greater influence. Conversely, within the KT-YOLO architecture that already possesses robust feature extraction capability, SlideLoss’s contribution is relatively modest but remains important, providing crucial final enhancement to overall performance.

**Fig 6:**
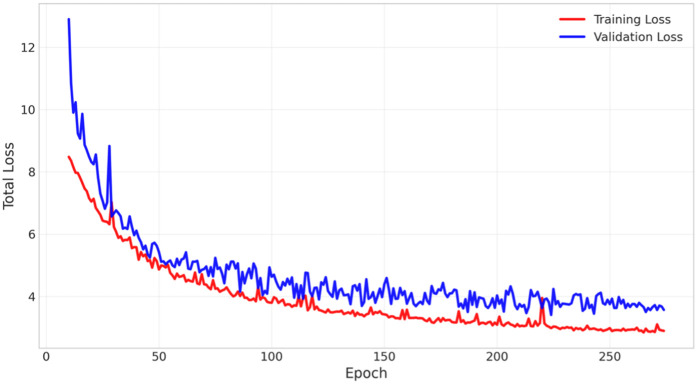
Training and Validation Loss of KT-YOLO. The loss curves demonstrate stable convergence throughout the training process with early stopping triggered at epoch 274, showing excellent generalization capability without overfitting.

The training and validation loss curves presented in Fig 6 further validate the robustness of the KT-YOLO architecture throughout the training process. The training loss and validation loss curves maintained close alignment from the 10th to the 274th training epoch, indicating that the model possesses excellent generalization ability without obvious overfitting phenomena. The early stopping mechanism was triggered at the 274th epoch (patience set to 50), confirming effective convergence of model training. The loss function demonstrated smooth decline and stable convergence during training, providing robust technical assurance for reliable deployment of KT-YOLO in actual agricultural environments. This stable training dynamics also indirectly validates the rationality of the kernel-team fusion mechanism and SlideLoss design. The systematic analysis of ablation experiments combined with the robustness verification of the training process collectively demonstrate the effectiveness and reliability of the KT-YOLO architecture for dense Hu sheep behavior detection tasks.

### Performance analysis

We evaluated the performance of models trained on the complete HSBD dataset under different lighting conditions to address the demands for all-weather monitoring in actual farming environments. As detailed in Table 2, the test set was categorized by capture time into daytime and nighttime subsets, utilizing the same trained model to perform detection tests on both subsets to verify the model’s lighting adaptability and robustness.

**Fig 7:**
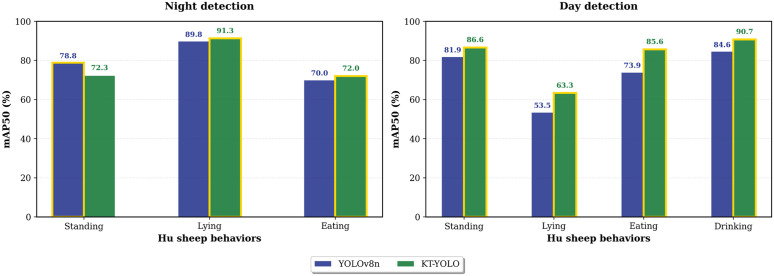
Behavior-specific detection performance under day and night conditions. Comparative analysis showing the detection accuracy of KT-YOLO versus YOLOv8n for standing, lying, eating, and drinking behaviors during daytime and nighttime periods.

Fig 7 presents the comparative performance of KT-YOLO and YOLOv8n across four Hu sheep behaviors under day and night conditions. The results demonstrate improvements in most behavior-lighting combinations.

**Daytime detection:** KT-YOLO achieved superior performance in all four behaviors: standing (86.6% vs 81.9%), lying (63.3% vs 53.5%), eating (85.6% vs 73.9%), and drinking (90.7% vs 84.6%). The most significant improvement was observed in lying behavior (+9.8 percentage points) and drinking behavior (+6.1 percentage points).

**Nighttime detection:** KT-YOLO maintained advantages in lying (91.3% vs 89.8%) and eating (72.0% vs 70.0%). For standing behavior, YOLOv8n achieved higher performance (78.8% vs 72.3%). Notably, lying behavior achieved the highest accuracy (91.3%) under nighttime conditions. Drinking behavior analysis was limited to daytime conditions, as Hu sheep exhibit minimal nocturnal drinking activity consistent with their natural circadian behavioral patterns. Supporting this observation, previous research has demonstrated that 84% of drinking events in sheep occur during daytime hours, with drinking frequency exhibiting a distinct 24-hour circadian rhythm and peak activity occurring around 10:54 h [32]. This diurnal drinking pattern reflects the inherent circadian regulation of water intake behavior in sheep. The few nighttime drinking instances observed were insufficient for meaningful statistical comparison.

The performance differential between day and night conditions reveals distinct patterns influenced by natural Hu sheep behavioral patterns. During daytime, Hu sheep exhibit greater behavioral diversity with frequent transitions between standing, eating, and lying behaviors, creating complex detection scenarios with increased inter-behavior occlusion and morphological similarities. The challenging distinction between low-head standing and actual eating behavior is particularly pronounced during active daytime periods.

Conversely, nighttime conditions naturally reduce behavioral complexity. Hu sheep predominantly adopt lying postures during rest periods, significantly reducing drinking frequency and eating activity. This behavioral concentration creates several detection advantages: (1) reduced behavioral diversity decreases morphological confusion between different postures, (2) clearer identification of standing individuals due to their rarity against the predominantly lying population, and (3) significantly reduced standing-eating confusion since nighttime feeding is minimal.

The experimental results validate these analyses: lying behavior achieved good nighttime performance (KT-YOLO: 91.3%, YOLOv8n: 89.8%). Standing behavior showed decreased detection accuracy at night (KT-YOLO: 72.3% vs 86.6% daytime), while eating behavior maintained reasonable performance under nighttime conditions (72.0% vs 85.6% daytime).

The consistent performance advantages stem from the kernel-team fusion mechanism’s multi-scale feature extraction capability. The combination of different convolution kernel sizes (1×1, 3×3, 5×5, 7×7) enables the model to capture both fine-grained individual features and broader spatial context information. This multi-scale approach proves particularly effective in dense scenarios where traditional single-scale methods struggle with occlusion and feature confusion.

KT-YOLO demonstrates consistent improvements in most behavior-lighting combinations, with the most significant gains observed in eating behavior during daytime (11.7% improvement) and lying behavior during daytime (9.8% improvement).

**Fig 8:**
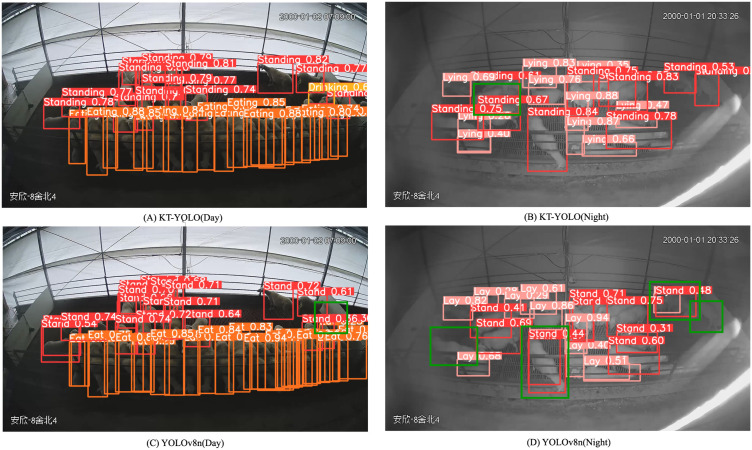
Detection results comparison between KT-YOLO and YOLOv8n. Red boxes: standing; pink boxes: lying; orange boxes: eating; yellow boxes: drinking; green boxes: detection errors (false positives and misclassifications). KT-YOLO demonstrates superior performance with more accurate detections and fewer errors.

These results indicate that KT-YOLO can provide reliable detection across varying environmental conditions while maintaining computational efficiency (5.8MB, 8.5 GFLOPs). Fig 8 provides qualitative visualization of detection results, illustrating the superior performance of KT-YOLO compared to YOLOv8n in both daytime and nighttime scenarios, with fewer missed detections and reduced false positives across different behavioral categories.

Despite the overall improvements, examination of the detection results reveals a notable failure case that merits discussion. In the nighttime scenario (Fig 8(B)), KT-YOLO produced a misclassification in the left region of the image, where overlapping individuals exhibiting lying and standing behaviors were present. KT-YOLO failed to correctly distinguish between them, whereas YOLOv8n successfully detected both the lying and standing individuals at the corresponding position in Fig 8(D). This failure can be attributed to the interaction between occlusion severity and reduced nighttime visibility. Under low-illumination conditions, the contrast between adjacent individuals diminishes significantly, and when two sheep with different postures overlap, the larger convolution kernels (5×5, 7×7) in the KTF module may aggregate features across individual boundaries, blurring the distinction between the occluded standing and lying postures. In contrast, YOLOv8n’s standard convolution operations, while generally less effective in dense scenarios, may preserve sharper local feature boundaries in this particular case. This observation indicates that while the multi-scale kernel-team fusion mechanism substantially improves overall detection performance in dense scenarios, handling extreme occlusion under low-visibility conditions remains a challenge that warrants further investigation.

### Seasonal performance analysis

To address the inherent influence of seasonal variations on animal behavior detection, we conducted a comparative analysis of KT-YOLO’s performance between summer (August 2023) and winter (January 2024) test subsets. Table 7 presents the per-behavior AP50 results across the two seasons using the same trained model.

**Table 7:** Seasonal comparison of per-behavior AP50 (%) for KT-YOLO.

Season	Standing	Lying	Eating	Drinking
Winter	87.1	85.6	85.2	92.1
Summer	86.6	71.0	85.6	92.2

The results reveal a notable seasonal disparity in lying behavior detection, with winter AP50 (85.6%) substantially exceeding summer performance (71.0%), representing a 14.6 percentage point difference. In contrast, standing, eating, and drinking behaviors exhibited minimal seasonal variation (within 0.5 percentage points), demonstrating robust cross-season generalization for these behavioral categories.

**Fig 9:**
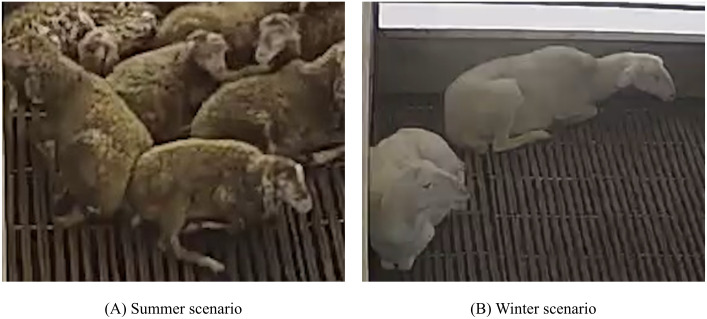
Seasonal visual comparison of Hu sheep farming scenarios. A: Summer scenario (August 2023) showing low foreground-background contrast, where Hu sheep fleece color closely resembles the pen floor, particularly affecting detection of lying individuals. B: Winter scenario (January 2024) showing high foreground-background contrast with clearly distinguishable individual contours against the background.

This performance gap in lying detection is primarily attributable to seasonal changes in visual appearance rather than behavioral pattern differences. As illustrated in Fig 9, summer conditions present substantially reduced foreground-background contrast: Hu sheep fleece becomes darker and visually similar to the pen floor due to accumulated soiling and environmental factors, while winter conditions maintain clearer contrast between the characteristically white fleece and the background. Lying behavior is disproportionately affected because the recumbent posture maximizes body contact with the ground surface, causing the sheep’s outline to merge with the background when contrast is low. Standing, eating, and drinking behaviors involve upright postures that maintain spatial separation from the ground plane, rendering them more resilient to contrast degradation.

These findings indicate that seasonal variation in visual conditions represents a meaningful factor affecting detection performance in intensive farming environments, and suggest that future work on appearance normalization or domain adaptation techniques could further improve cross-season robustness, particularly for recumbent posture detection.

## Discussion

This study constructed the Hu Sheep Behavior Dataset (HSBD) and proposed KT-YOLO for automated Hu sheep behavior detection in dense farming environments. HSBD contains 280 images with 6,766 Hu sheep instances, with up to 36 sheep per image. KT-YOLO integrates the kernel-team fusion mechanism and SlideLoss loss function, effectively addressing detection challenges in dense farming scenarios through multi-scale feature extraction and class balance optimization while maintaining excellent computational efficiency. Experimental results demonstrate that KT-YOLO achieved 86.4% mAP50, representing a 6.3% improvement over the baseline YOLOv8n model, with the model exhibiting favorable accuracy-efficiency trade-offs through a compact model size of 5.8MB, computational requirements of 8.5 GFLOPs, and an inference speed of 85.3 FPS. The mAP50:95 of 60.8% further confirms robust localization quality at stricter IoU thresholds.

The superior performance of KT-YOLO can be attributed to several key innovations. The kernel-team fusion mechanism addresses the fundamental challenge of multi-scale feature extraction in dense scenarios [23] by employing four different convolution kernel sizes simultaneously. This design enables the model to capture both fine-grained individual characteristics and broader contextual information, which is crucial for distinguishing behaviors in crowded environments where individual sheep may be partially occluded.

The integration of SlideLoss function effectively addresses the inherent class imbalance problem in livestock behavior datasets. Natural animal behavior patterns result in unequal distribution of different activities [8,9], with some behaviors like drinking occurring much less frequently than others like standing or lying. SlideLoss dynamically adjusts the attention given to different samples based on their difficulty, ensuring that the model learns to recognize all behaviors effectively rather than being biased toward the most common ones.

The experimental results demonstrate clear advantages of our approach across multiple evaluation dimensions. Compared to established methods like Fast R-CNN [34] and Swin Transformer [35], KT-YOLO achieves significantly higher accuracy while maintaining much lower computational requirements. This efficiency is crucial for practical deployment in agricultural environments where edge computing resources may be limited [24].

Beyond the specific detection challenges identified in the results (e.g., occlusion-related misclassification under nighttime conditions), this study acknowledges several broader limitations. First, all 280 images in HSBD originate from a single commercial farm with fixed camera installations. To mitigate temporal redundancy, frames were sampled at 180-frame intervals, and two collection periods covering summer 2023 and winter 2024 introduce natural variation in illumination and flock distribution. Nevertheless, the limited image count and single-site origin may constrain the generalizability of the findings to other farming environments. Second, although the seasonal analysis (Table 7) revealed meaningful performance variation, particularly the 14.6 percentage point reduction in lying detection AP50 during summer attributable to reduced foreground-background contrast, the limited number of images per season precludes definitive conclusions regarding seasonal generalizability. Future studies with larger per-season sample sizes would enable more robust characterization of seasonal effects on detection performance. Third, the model was developed and validated exclusively on Hu sheep, which possess uniformly white fleece and medium body size. The applicability to breeds with different coat colors (e.g., Suffolk), varying stocking densities, or different camera installation configurations remains to be empirically validated.

## Conclusion

This paper presents KT-YOLO, a novel deep learning architecture specifically designed for dense Hu sheep behavior detection in intensive farming environments. The main contributions include the development of the HSBD dataset containing 6,766 sheep instances across 280 images, the innovative KTF mechanism for multi-scale feature fusion, and the integration of SlideLoss to address class imbalance challenges.

Experimental validation demonstrates that KT-YOLO achieves 86.4% mAP50, representing a significant 6.3 percentage point improvement over the baseline YOLOv8n while maintaining computational efficiency with only 5.8MB model size, 8.5 GFLOPs, and 85.3 FPS inference speed, achieving 60.8% mAP50:95 for precise localization. The model shows robust performance across different lighting conditions and behavioral categories, demonstrating promising potential for continuous monitoring in intensive Hu sheep farming environments, with further cross-farm and cross-breed validation warranted.

Future work should prioritize the following research directions. First, validate the transferability of KT-YOLO across different sheep breeds and livestock species, across farming facilities with varying camera configurations, and toward additional behavioral categories beyond the four basic behaviors examined in this study. This should evaluate the cross-domain adaptation capability of the multi-scale feature fusion mechanism. Second, integrate temporal modeling approaches such as LSTM-based sequence analysis to capture behavioral transitions and temporal dependencies, which would also enable investigation of seasonal behavioral variation across different environmental conditions. Third, the seasonal performance disparity observed in lying behavior detection (Table 7) suggests that appearance normalization or domain adaptation techniques warrant investigation to improve cross-season robustness under varying visual conditions.

## Supporting information

S1 Fig Representative raw video frames from HSBD under daytime and nighttime conditions. Unannotated frames depicting the typical daytime (A) and nighttime (B) scenarios in the Hu sheep barn at Anxin Animal Husbandry Co., Ltd., Bozhou City, Anhui Province, China. These frames correspond to the raw visual inputs from which the detection results presented in Fig 8 were generated, and are provided for reference to illustrate the original scene appearance prior to model inference. Both images are original to this work and were captured during the data collection periods described in Materials and Methods.
(TIF)
