# Supplementary figures and images for: KT-YOLO: A multi-convolution kernel collaboration model for dense Hu sheep behavior detection
Source: PLoS One. 2026 May 18;21(5):e0349267. doi: 10.1371/journal.pone.0349267 (PMC13183238; doi:10.1371/journal.pone.0349267)

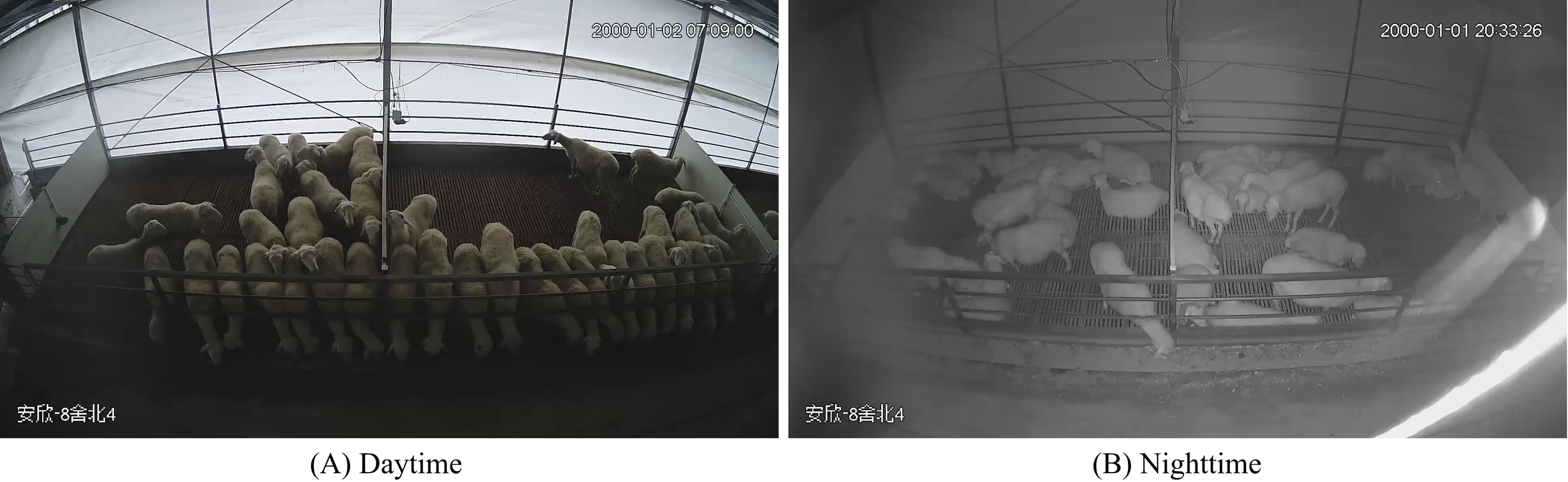

Supplement: S1 Fig — Unannotated frames depicting the typical daytime (A) and nighttime (B) scenarios in the Hu sheep barn at Anxin Animal Husbandry Co., Ltd., Bozhou City, Anhui Province, China. These frames correspond to the raw visual inputs from which the detection results presented in Fig 8 were generated, and are provided for reference to illustrate the original scene appearance prior to model inference. Both images are original to this work and were captured during the data collection periods described in Materials and Methods. (TIF) [file pone.0349267.s001.tif]
